# RETRACTION: Comparison of the Risk of Postoperative Wound Infection in Patients With Rectal Cancer by Laparoscopic Versus Open Hartmann's Surgery

**DOI:** 10.1111/iwj.70403

**Published:** 2025-03-21

## Abstract

**RETRACTION:** JiangY. , LiuR. , YouQ. , FanX. , WuY. , and ZengZ. , “Comparison of the Risk of Postoperative Wound Infection in Patients With Rectal Cancer by Laparoscopic Versus Open Hartmann's Surgery,” International Wound Journal 21, no. 2 (2024): e14752, 10.1111/iwj.14752.PMC1085079242052894

The above article, published online on 07 February 2024, in Wiley Online Library (http://onlinelibrary.wiley.com/), has been retracted by agreement between the journal Editor in Chief, Professor Keith Harding; and John Wiley & Sons Ltd. Following an investigation by the publisher, all parties have concluded that this article was accepted solely on the basis of a compromised peer review process. The editors have therefore decided to retract the article. The authors did not respond to our notice regarding the retraction.

**RETRACTION:** Y. Jiang , R. Liu , Q. You , X. Fan , Y. Wu , and Z. Zeng , “Comparison of the Risk of Postoperative Wound Infection in Patients With Rectal Cancer by Laparoscopic Versus Open Hartmann's Surgery,” International Wound Journal 21, no. 2 (2024): e14752, 10.1111/iwj.14752.PMC1085079242052894

The above article, published online on 07 February 2024, in Wiley Online Library (http://onlinelibrary.wiley.com/), has been retracted by agreement between the journal Editor in Chief, Professor Keith Harding; and John Wiley & Sons Ltd. Following an investigation by the publisher, all parties have concluded that this article was accepted solely on the basis of a compromised peer review process. The editors have therefore decided to retract the article. The authors did not respond to our notice regarding the retraction.

